# Supplementary material for: T-BACCO SCORE: A predictive scoring tool for tuberculosis (TB) loss to follow-up among TB smokers
Source: PLoS One. 2023 Jun 15;18(6):e0287374. doi: 10.1371/journal.pone.0287374 (PMC10270618; doi:10.1371/journal.pone.0287374)
Supplement: Attachment — Submitted filename: Response to Reviewers.docx [file pone.0287374.s001.docx]

Response to Reviewers

Dear PLOS One Editors,

AMENDMENT TO THE MANUSCRIPT ENTITLED “T-BACCO SCORE: A PREDICTIVE SCORING TOOL FOR TUBERCULOSIS(TB) LOSS TO FOLLOW-UP AMONG TB SMOKERS

|  | Major Concerns |  |
| --- | --- | --- |
| 1 | There is no reporting of how well the model performed in the internal validation cohort. | Reports on the performance of the internal validation cohort is available in the results section under the performance of final model. |
| 2 | The HOsmer-Lemeshow is not a reliable test for calibration, authors should present calibration plots as illustrated in the TRIPOD statement for reporting of risk prediction models. | As per suggestion, calibration plots have been added in addition to the Hosmer-Lemeshow value for the model calibration reporting. The calibration curve for the final model and the internal validation model are available in Fig 3 and Fig 4. |
| 3 | Early in the paper it is not clearly and consistently articulated that a simple binary indicator of 'loss to follow-up (LTFUP)' is the outcome being modeled. It is confusing with all the information presented on different outcome states. is the outcome LTFUP relative to the occurrence of all other outcome types? | The outcome being measured and modelled in this study is the LTFU. The LTFU outcome is being compared with the successful TB outcome (cured and completed treatment) |
| 4 | Cross-sectional: the study uses fixed, baseline values of predictors to calculate the occurrence of LTFUP, which happen at some point in the future, so the repeated use of cross-sectional seems not quite accurate | We have look back at the methods of this study. We agree that the term “cross-sectional study” used in this study is inappropriate. This study involves secondary data analysis of a cohort of TB patients from year 2013-2017 in the national TB database. We looked back at the predictors that lead to the LTFU outcome. Thus, the correct study design for this study would be a retrospective cohort study. |
| 5 | On line 29 no statement about how much data is missing, whether it can be assumed to be missing at random, and whether any form of imputation was used. If not, how was this justified? | Additional statement on the missing data information has been added in the abstract and result section. We have run the missing value analysis and the little MCAR test to report on the data missingness. Certain variable in the database has been recategorized and subsequent changes on the results and figure has been amended. |
| 6 | Process of multivariable model selection should be in the stats section rather than in results. | The paragraph on variable selection for the final model has been changed to the methodology section under the statistical analysis part. |
| 7 | On line 188 test of equality for two AUC values should use DeLong's test rather than chi-square. | As per suggestion we have analyse the performance between the two-model using the DeLong’s test. The outcome of this test is included under the result section in the performance of the final model paragraph. |
| 8 | The tiny fraction of women in the study suggests these results should be limited to males only. | The prevalence of woman who smokes in our country based on the latest national health morbidity survey (NHMS, 2015) is 1.3% which correspond to our finding in this study. Thus, we choose to remain the female subject in this study. Besides that, gender was not a significant predictor in this study, therefore the small proportion of female gender do not affect the performance of the model. |

|  | Minor Comments |  |
| --- | --- | --- |
| 1 | Multivariable instead of multivariate when modeling a single outcome. | The term multivariate used in this manuscript has been changed to multivariable throughout the manuscript. |
| 2 | AUC provides fair, not good, discrimination. | Correction has been made. The term used on the AUC has been changed to fair discrimination. |
| 3 | Line 134 confusing. | The sentence has been rearranged. The outcome being measured in this study is the LTFU TB outcome. |
| 4 | Lines 187-8, chi-square not appropriate for comparing two AUC. | As per suggestion we have analyse the performance between the two-model using the DeLong’s test. The outcome is available under the result section. |
| 5 | line 220: need to define RM2160 which presumably is transparent to Malaysians. | The categorisation used for the income level is according to the median personal income for the population of Selangor state based on the department of statistic Malaysia (DOSM, 2018). Details on this variable has been added in line 187 under the statistical analysis paragraph. |
| 6 | Line 244 - state 14 variables retained but only 13 listed. | We have corrected the sentence. The correct number of variables included in the multivariable analysis were 14, and 12 were retained in the final model. |
| 7 | line 260: no mention of model performance in validation cohort. | The performance of the Internal validation model was mentioned under the result section in the performance of the final model paragraph. |
| 8 | lines 282-3: no comprehensive discussion or treatment of missingness | Management and reports on the missing data has been included in the result section under the prognostic scoring system for LTFU paragraph. |
| 9 | lines 288-9: that chi-square test does suggest anything about missing values being completely at random. | We have conducted the missing value analysis and the little’s MCAR test to handle the missing data in the database. The report on the chi-square test has been corrected. |
| 10 | Table 5: okay to present Hosmer-Lemeshow as supplement to calibration plots. | Calibration curve for both final and internal validation model has been plotted under Fig 3 and Fig 4 in the manuscript using R statistical package. |
| 11 | line 351: need to define IVDU | IVDU has been defined as intravenous drug user in the sentence. |
