# Supplementary material for: T-BACCO SCORE: A predictive scoring tool for tuberculosis (TB) loss to follow-up among TB smokers
Source: PLoS One. 2023 Jun 15;18(6):e0287374. doi: 10.1371/journal.pone.0287374 (PMC10270618; doi:10.1371/journal.pone.0287374)
Supplement: Attachment — Submitted filename: Response to Reviewers.docx [file pone.0287374.s002.docx]

Response to Reviewers

Dear PLOS One Editors,

AMENDMENT TO THE MANUSCRIPT ENTITLED “T-BACCO SCORE: A PREDICTIVE SCORING TOOL FOR TUBERCULOSIS(TB) LOSS TO FOLLOW-UP AMONG TB SMOKERS

Response to Reviewer 1:

|  | Comments | Response |
| --- | --- | --- |
| 1 | On line 51 put a comma in 835,000 | Correction has been made accordingly on-line 51 |
| 2 | Revise line 178 as follows: ' ... with LTFU were analysed using ... ' | The sentence has been corrected on line 178 “with LTFU were analysed using a simple logistic regression analysis”. |
| 3 | Revise line 183 as ' For the monthly income level, the cut point at ... (488.30 USD) was based on ... ' | The cut of point at RM2160 (488.30 USD) used was based on the median personal income of population in this country according to the department of statistics Malaysia in 2018 (DOSM). |
| 4 | Line 227: homogeneous | Spelling correction has been made accordingly, from “homogenous” to “homogeneous”. |
| 5 | In Fig 2 please add the labelled AUC for each curve. | Amendment has been made in Fig 2. AUC value for each curve has been added. |
| 6 | For Fig 3 recommend confidence limits for blue model performance to show overlap with diagonal. | Calibration curve Fig 3(A) and Fig 3(B) has been amended. The confidence limits each calibration curve was inserted as per-recommendation. |

Response to Reviewer 2:

|  | Comments | Response |
| --- | --- | --- |
| 1 | The outcome is loss to follow-up. What’s the length of follow-up time for each individual? Are they very different or the same across the individuals? If vary, will this confound the results? | The length of follow-up time unlikely confound the results. Majority of each individual follow-up duration is limited to the recommended six-month of TB treatment duration. Patients with TB MDR status (who needs longer treatment duration) has been excluded from the databased. |
| 2 | Line 179. The significance level was set to be 0.025. Please explain why this threshold is selected? | For variable selection into the multiple logistic regression analysis, significance level was set at 0.25 (not 0.025). This threshold was selected to ensure that adequate variable can be considered in the final logistic model. |
| 3 | Line 194. AUC of 0.5 is selected as a discrimination threshold. However, 0.5 is just the value with random classification. Will such a low threshold be a concern? | We are aware that AUC of 0.5 indicates no discrimination and AUC 0.7 is considered acceptable. However, in clinical setting within the TB domain especially on LTFU issue an AUC more than 0.6 is considered satisfactory as compared to the common acceptable threshold of 0.7. We have revised the sentence in line 194 to “AUC value ranges from 0.5 to 1, where a greater AUC value indicates that the model has better ability to distinguish patients who were LTFU and had successful TB treatment outcome”. |
| 4 | Line 199. De Long test was used to compare the AUC between models. Please clarify what two models were compared? development and the internal validation cohort? If so, does it really make sense to compare their AUC when they are using different data? | Thank you for your comment. Yes, the two model that were compared are the development and the internal validation cohort. Initially, De Long test was used to inform that the performance between the two model has not much difference. However, after careful reading, we realized that De Long test is used to compare two correlated model. Therefor this analysis will be removed from this study finding. The comparison of the ROC between the two cohort will be assessed based on their overlapping confidence interval:  Development cohort: 0.681 (95% CI 0.657-0.713)  Internal validation cohort: 0.668 (95% CI 0.639-0.698) |
| 5 | It’s good to see the missingness doesn’t differ between development cohort and internal validation cohort. However, shouldn’t the missing data be taken care before split the sample into two cohorts? | We agreed that missing data analysis should be managed earlier, however the two cohort (development and the internal validation) are coming from the same source of data based and it was divided randomly, therefor it should not be a concern. The purpose of analysing the missing data separately is to provide a more representative picture on the data missingness for each cohort to the reader. |
